# The Anticipation of Crime and Corruption Problems Due to the Expansion of Telemedicine: A Study Based on the Korea Medical Crime Investigation System

**DOI:** 10.3389/fpubh.2021.767671

**Published:** 2021-11-08

**Authors:** Lee Dongkyu

**Affiliations:** Police Human Resource Development Institute, Asan, South Korea

**Keywords:** telemedicine, telehealth, medical crime, medical corruption, COVID-19

## Abstract

The telemedicine system, which has been gradually introduced, has changed dramatically with the outbreak of COVID-19. Now, with the development of related laws and technologies, the introduction of telemedicine will be further accelerated, and like the advent of smartphones, this will become an unstoppable trend of the times. However, just as there are various crimes and corruption problems in the current health system, the introduction of telemedicine may bring other problems. Therefore, it is important to anticipate the types of corruption or crimes that will occur with the introduction of telemedicine. And based on these expectations, we will have an opportunity to properly prepare for the various problems associated with telemedicine.

## Introduction

Many people consider themselves to be in the middle of changes. In just a few decades, we have taken e-mail and smartphones for granted, and social media has become a part of our lives. The way we live, learn and connect with people is also changing dramatically. It is so natural for people to move from LTE to 5G now that they do not even feel that it is a big change. However, despite these rapid technological changes, there have been some areas that did not change. Education at school, meetings, and hospital treatment have not changed surprisingly despite the rapid change in technology. Educational methods and treatment methods have maintained the face-to-face method of students and teachers and patients and doctors despite advances in related technologies. Of course, with the development of information and communication technology, even though non-face-to-face methods have been attempted in fields such as education and medical care, many people still think that it is somewhat inconvenient and uncertain, so it has not been successfully introduced.

However, the SARS-CoV-2 pandemic has dramatically changed these circumstances. Many schools have rapidly introduced online classes, and many conferences, including those between heads of state, have been turned into cyberconferences. Traditional medical methods have also been required to change. Of course, there have been discussions (1) or progress on telemedicine in many countries previously. Many countries, including the United States (2), Australia, Europe, China, Japan, and Korea, have prepared or carried out telemedicine, but the coronavirus pandemic has demanded a more rapid introduction of this technology. COVID-19 has radically changed the adoption of telemedicine, and in futurology, these unexpected and big drivers of change are called wild cards (3).

### COVID-19 and Telemedicine

There has been a lot of discussion about telemedicine in the past. In the case of Korea, the concept of telemedicine, which uses a computer to diagnose a patient at a distance, appeared in 1976 (4), and since 1991, the telemedicine system has been piloted for residents in areas where hospital treatment is difficult. The government wanted to actively introduce a telemedicine system, but doctors have consistently opposed it, and for this reason, telemedicine was very limited before the COVID-19 pandemic.

However, after the COVID-19 pandemic, telemedicine was temporarily allowed in Korea from February 24, 2020 to protect medical staff, prevent group infection, and overcome the shortage of medical staff. From February 24, 2020, to July 4, 2021, the number of telemedicine sessions, including telephone treatment, reached 2,378,510, in the order of internal medicine, neurology, psychiatry, and obstetrics and gynecology with an average of 100,000 to 200,000 telemedicine sessions per month (5).

In the case of the United States, which has been proactive in introducing telemedicine in the past, the rate of telemedicine service usage, which was only 11% before the COVID-19 pandemic, increased to 46%, and the use of telemedicine by doctors and medical institutions also increased by 50 to 175 times. Some experts estimate that up to $250 billion of US healthcare spend could potentially be shifted to virtual or virtually enabled care (6).

In the UK, the number of telemedicine cases is rapidly increasing, with 5.4 million users using services such as Push Doctor (https://www.pushdoctor.co.uk/) (7), one of the telemedicine services, and the situation in France and other European countries is similar. In China, more than 400 million users have signed up for telemedicine services such as Ping An Good Doctor (http://www.pagd.net/), and the cumulative number of users has already exceeded 1.1 billion (8).

### Acceleration of the Era of Telemedicine

The accelerated use of telemedicine due to the COVID-19 pandemic (9, 10) is a global trend, and it is highly likely that this trend will not change any time soon in the future for the following reasons.

#### Convenience

As in the case of online video conferencing, telemedicine is likely to be uncomfortable for both patients and doctors in the beginning. However, once you get used to the method, you can have a much higher level of convenience than the general face-to-face treatment in terms of time and cost. People who have experienced this convenience once will not be able to return to the previous treatment method again. As seen in many technological cases such as smartphones, people do not give up the technological convenience once they experience it for the first time, and this concept also applies to telemedicine.

#### Cost

Although there may be differences between countries, in general, telemedicine is cheaper than general medical care, which can be a huge advantage. From the point of view of medical institutions, it is highly likely to reduce additional costs in the long run, such as waiting for patients, space for examination, and cost of management. The number of patients that can be treated per hour is likely to increase. Therefore, telemedicine can generate economic benefits for both patients and doctors in the long run.

#### Technology Development

Smart watches used by many people already obtain simple health information. They are equipped with electrocardiogram, heart rate, and blood oxygen saturation functions and also provide warnings about arrhythmias and measures the quality of sleep. Smartwatches will also measure blood sugar. As wearable devices such as smartwatches continue to develop technologically, the related market size is expected to continue to grow. In addition to wearable devices such as smart watches, full-scale telemedicine devices are continuously developing. Smaller and more precise telemedicine devices are being developed and distributed, and the development of communication technology and VR technology is also supporting this (11). Therefore, the use of telemedicine or medical applications using these telemedicine devices will increase rapidly.

## Corruption and Crime Anticipation in Telemedicine

**Figure 1:**
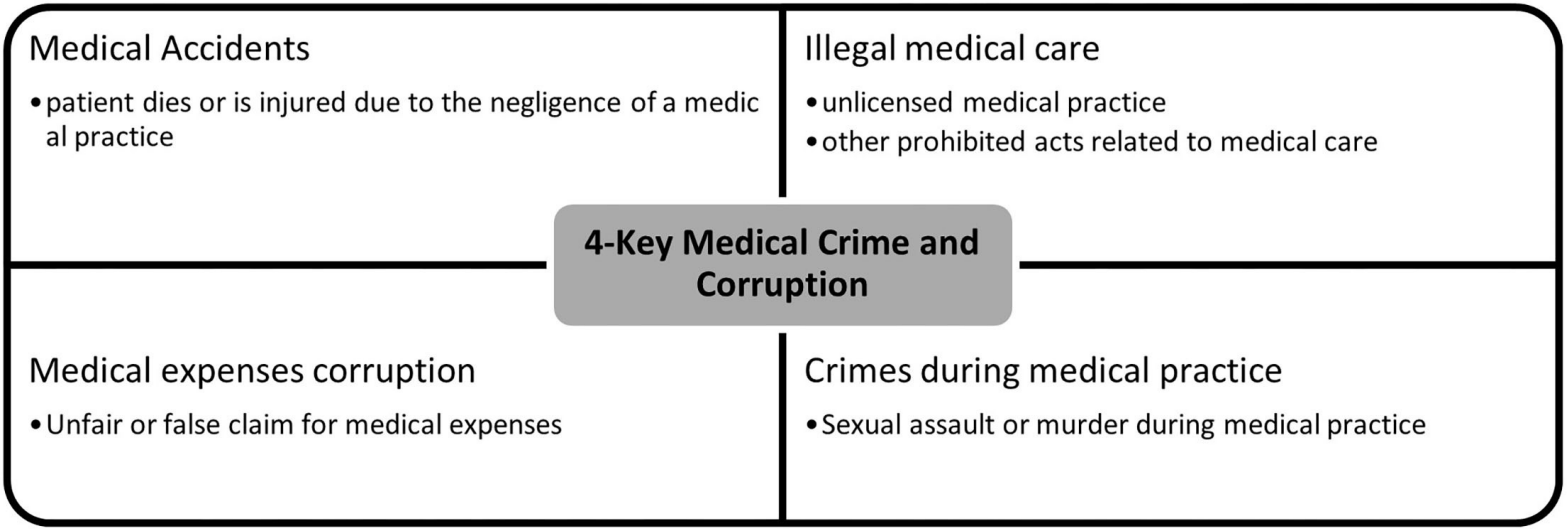
4-Key Medical Crime and Corruption.

To discuss corruption and crime in telemedicine, it is necessary to first review the current medical crime and corruption issues. Medical care generally requires very technical and highly specialized knowledge, and because the scale of the medical industry in modern society is very large, there are various crimes and corruption related to it. Medical crime investigations in Korea are centered on the following four issues (12). The four most important issues related to medical crime or corruption are shown in the Figure 1 below.

### Four-Key Medical Crime and Corruption Issues

#### Medical Accidents

These accidents refer to the kind in which a patient dies or is injured due to the negligence of a medical practitioner during medical treatment. Medical accidents are one of the oldest and most sensitive problems related to medical care, and there are reports that more than 200,000 people die each year in the United States. In the case of physical damage caused by medical treatment, there are cases that are resolved through individual lawsuits or punished as a crime.

#### Illegal Medical Care

It refers to general prohibited acts related to medical care, such as unlicensed medical practice. In this Regard, Illegal Drug Prescription and use, Unlicensed Medical Treatment, False Advertising, Drug Rebate, etc. may apply. These vary according to each country's legislation, but unlicensed medical practice is mostly a crime subject to punishment.

#### Medical Expenses Corruption

This type of corruption refers to unfair or false claims for medical expenses. This may be a case when a patient makes an unfair claim to an insurance company, or a medical institution makes an unfair claim to a health insurance institution. In general, it refers to a case in which a medical institution makes an unfair claim for payment to a health insurance institution. Some studies have shown that these medical scams have a very negative impact on medical finances (13), but they are often ignored. This medical fraud problem is one of the important problems occurring worldwide.

#### Other Crimes During Medical Practice

Other crimes may occur during medical practice, such sexual assault on a patient, intentional injury, or murder under the guise of medical practice.

### Review of the Current Telemedicine System

#### Definition of Telemedicine

In the case of telemedicine, a conceptual definition is needed. Telemedicine does not have an exact definition at present, and various terms such as telehealth and telemedicine tend to be used interchangeably (14), and recently, the terms non-face-to-face treatment (medicine), smart treatment, u-health, e-health, and virtual treatment have also been used. Although the above terms have similar contents, there are also differences, and the aim of each term is different. An important concept among these is the concept of telemedicine and telehealth, which differs from country to country, but the general definition is as follows (15).

##### Telemedicine

It means that a doctor treats a patient and provides medical services by means of telecommunications without the patient and the doctor being in the same place. Generally, telemedicine can only be performed by a licensed physician.

##### Telehealth

In addition to telemedicine, it means providing comprehensive health services such as a variety of non-physician services, including telenursing and telepharmacy. Some of these services may be provided by persons without a medical license.

In other words, telemedicine generally refers to medical services such as a diagnosis received at a hospital, and telehealth includes education and comprehensive health management in addition to telemedicine.

This study focused on telemedicine performed by doctors rather than telehealth as a comprehensive concept.

#### Current Telemedicine Issues

Telemedicine is expected to occur and take place in many ways, and many countries are trying to introduce it. It is a welcomed technology because it is possible to provide services to areas where it is difficult to obtain medical services, and it increases convenience for existing patients. However, telemedicine is currently facing problems from several perspectives.

Although it varies from country to country, in the case of telemedicine, legal issues are often pointed out first. Because the current medical system or law is based on direct patient-doctor contact, telemedicine can be subjected to legal regulations in each country (16). There is also the question of whether a medical license recognized in a particular state or country can be recognized in other regions (17). However, the coronavirus pandemic has caused these restrictions to be either rapidly eased or temporarily removed and has shown that legal obstacles can be overcome. The fact that telemedicine has temporarily expanded significantly, but no major problems have been reported so far supports the argument that these legal regulations should be relaxed. In the future, there will be a lot of discussion about the qualifications of telemedicine, whether a medical license is recognized by region or country, and the scope and target of telemedicine.

From a technical point of view, there may be problems with equipment for telemedicine. Unlike general medical devices, there are no international standards for medical devices that can be used for telemedicine, and their reliability has not been verified. In some studies (18), the reliability of these telemedicine devices has been studied, and it will be necessary to continuously review such international standards in the future.

#### Features of Telemedicine

Telemedicine has several characteristics that are different from traditional medical practices, and these characteristics must be identified first to review possible corruption or illegal activities related to telemedicine.

##### Use of Telecommunication Equipment

Telemedicine is essentially based on telecommunication equipment (19). Telecommunication equipment may include everything from general telecommunication equipment such as telephones and video calls to professional medical equipment such as telemedicine examination device.

##### Requirement of Authentication Means

Separate essential authentication means should be provided. Online authentication of doctors and patients is required, such as a doctor's license, medical insurance, and patient identity. In the case of telemedicine, this authentication must be done remotely.

##### Sensory Restrictions

Currently, telemedicine is limited to listening to a patient's statement, observing with a camera or monitor, or using a simple medical examination device. Therefore, touch and smell are blocked among the five senses, and hearing and sight are generally limited. In addition, in the case of body temperature, pulse, and blood sugar levels, the precision may be limited when the patients directly perform the test themselves.

### Anticipating Crimes and Problems Related to Telemedicine

Based on the current state of medical crime and corruption, if the characteristics of telemedicine are considered, it is possible to forecast future crimes and corruption that may occur through telemedicine. When telemedicine is implemented in earnest, the main 6 problems that may arise may include the following. These problems are predicted by reflecting the characteristics of telemedicine based on the existing system of medical corruption and crime.

#### Medical Payment Fraud

Medical institutions generally store the personal information of patients. If medical institutions abuse this, medical institutions may falsely request payment from health insurance payment institutions even though the actual patient did not receive treatment. These false claims have often been pointed out before, but when medical institutions store more personal information and telemedicine is activated, this weakness may become more apparent. This medical information can also be obtained by groups who hack data, but because only medical institutions can claim medical expenses, this is likely to happen mainly in medical institutions.

#### Medical Accidents Caused by Telemedicine

Due to the limitations of telemedicine, there is a possibility that medical accidents will occur. This is a case in which the symptoms of a problem are incorrectly diagnosed due to the limitations of telemedicine. This is one of the biggest reasons doctors are currently opposed to telemedicine. However, this risk may be limited because telemedicine is mostly limited to non-fatal diseases.

#### Unlicensed Medical Qualifications and Devices

Telemedicine has several advantages over the previous medical system in terms of cost, time, and accessibility, so there is a possibility that traditional unlicensed medical practice will decrease. On the other hand, other issues related to licensing may arise. Currently, there is a discussion about whether a doctor's license can be recognized in each state (17), and there may be cases where online platforms are used to see doctors from other countries. It can also open up unauthorized hospitals on places like the dark web. In some ways, these unlicensed online medical institutions may be easier to create and more difficult to detect than ever before. People who have difficulty visiting hospitals, such as criminals, can easily access these illegal services.

As telemedicine advances, various types of medical devices can be used. These devices must be reliable as an information and communication device and as a medical device. These medical devices are likely to be expensive. Accordingly, various problems related to telemedicine may occur, such as illegally manufacturing these medical devices, using unauthorized medical devices, and recycling broken medical devices. Additionally, these problems can occur not only in hardware but also in software such as mobile apps.

#### Abuse of Telemedicine Data

Telemedicine collects, transmits, and stores highly sensitive personal medical data such as videos, photos, and physical information. As telemedicine becomes active, such data will increase significantly, and there is a high possibility of sharing or using it beyond keeping it for each medical institution. Personal health information is one of the most sensitive and important personal data in modern society and can be a target for attack at any time. Companies such as Facebook, which have a high security level, are often attacked, and information is leaked, but the current security level of medical institutions is even more insufficient. In the US, over 500 healthcare providers suffered ransomware attacks in 2020 alone (20). As such, attacks on medical information will continue. In addition to being attacked by professional hackers, telemedicine information has many vulnerabilities. Some medical professionals may illegally sell or share this information elsewhere, while other medical professionals may personally use videos or photos of patients. In addition, considering the usefulness of these data, there is a possibility that it may be illegally used for commercial purposes.

#### Drug Abuse

In the case of telemedicine, it is usually very easy to purchase medicines. In Korea, a service that delivers related medicines to your home within 30 min after telemedicine is gaining popularity. This is a very convenient service, but in these cases, the drug is likely to be abused or not managed properly. After a simple telemedicine session, sensitive medicines such as erectile dysfunction drugs can be easily purchased or used. Restricting such purchasing methods may go against the basic purpose of telemedicine, so it is necessary to find an appropriate balance point. Some countries, such as China, are tightening regulations on online sales of drugs (21).

## Discussion

### Policy Recommendations

To prevent various problems expected with the introduction of telemedicine, institutional and technical preparations are needed.

Institutionally, it is necessary to reorganize regulations on telemedicine and make internationally standard regulations. Through these standard regulations, it will be possible to supplement and apply them according to the specific situation of each country. Establishing a procedure or method through an international standard such as ISO may be considered. Through the establishment of such international standards, the reliability of telemedicine will be improved, and countries that have not introduced telemedicine will be able to introduce telemedicine more easily. In addition, it may be easier to help countries in need of medical assistance. Technical preparation is also very important. Patients must have access to telemedicine through an official and trusted method, and healthcare providers must be able to accurately identify patients. To prevent corruption such as illegal medical bills, information on medical practices should be verified in real time. Through this, problems related to illegal medical expenses can also be prevented. In addition, special technical measures are required to protect individual patients and medical information, and medical institutions must assume the responsibility for these safeguards. Telemedicine devices should be made to ensure reliability by establishing technical standards.

In order to solve these institutional and technical problems, it is necessary to consider the establishment of a professional institution to manage them. Telemedicine is a complex mixture of technical problems along with the existing medical system, and there are currently no specialized institutions to prepare for and manage these problems. Therefore, it is necessary to consider the establishment of an institution or department that can comprehensively manage these problems.

### Research Limitations and Future Research Directions

The introduction of telemedicine has been made rapidly around the world due to the COVID-19 pandemic, and it is highly likely that it will become an unstoppable flow like the smartphone epidemic. However, just like cybercrime has been in the past, technological advances will inevitably lead to related crimes and corruption. Medical care is a very important value for people, and this study may be meaningful in terms of anticipating and preparing for possible problems related to telemedicine in the future.

However, this study is based on existing studies and has a limitation in that there are no specific cases or data related to telemedicine. In the future, if telemedicine becomes more active around the world and related cases and data are published in the literature, more research will be possible.

## Data Availability Statement

The original contributions presented in the study are included in the article/supplementary material, further inquiries can be directed to the corresponding author/s.

## Author Contributions

The author confirms being the sole contributor of this work and has approved it for publication.

## Conflict of Interest

The author declares that the research was conducted in the absence of any commercial or financial relationships that could be construed as a potential conflict of interest.

## Publisher's Note

All claims expressed in this article are solely those of the authors and do not necessarily represent those of their affiliated organizations, or those of the publisher, the editors and the reviewers. Any product that may be evaluated in this article, or claim that may be made by its manufacturer, is not guaranteed or endorsed by the publisher.
